# Growing teratoma syndrome with extra pelvic metastasis and gliomatosis peritonei

**DOI:** 10.1016/j.gore.2025.101731

**Published:** 2025-03-29

**Authors:** Brittany File, Sonia Lee, Robert Bristow

**Affiliations:** a Department of Obstetrics and Gynecology, University of California, Irvine Medical Center, Orange, CA, USA; b Department of Radiologic Sciences, University of California, Irvine Medical Center, Orange, CA, USA; c Department of Obstetrics and Gynecology, Division of Gynecologic Oncology, University of California, Irvine Medical Center, Orange, CA, USA

**Keywords:** Growing teratoma syndrome, Gliomatosis peritonei, Immature teratoma, Case report

## Abstract

• Growing teratoma syndrome occurs when mature teratomas are discovered in patients who have previously received adjuvant chemotherapy for immature teratoma.
• Gliomatosis peritonei is a rare condition often found alongside immature teratoma.
• Synchronous identification of growing teratoma syndrome and gliomatosis peritonei has rarely been reported.
• Comprehensive abdominopelvic imaging is critical to the surveillance protocol after treatment of immature teratoma to assess and appropriately treat this phenomenon.

## 1 Introduction

Ovarian immature teratoma is a rare form of ovarian cancer most frequently diagnosed in adolescents. It represents < 1 % of all ovarian carcinomas and generally has a favorable prognosis with an excellent overall survival rate utilizing adjuvant chemotherapy with bleomycin, etoposide, and cisplatin (Moraru et al., 2023).

Growing teratoma syndrome (GTS) is a rare occurrence in which mature teratoma components grow in the setting of adequately treated immature teratoma and tumor markers remain normal and stable with one study estimating an incidence of 12 % (Logothetis et al., 1982, Zagamé et al., 2006). Although benign, early identification of GTS is critical so as to exclude recurrent immature teratoma and perform debulking surgery while masses remain small to prevent mechanical complications and obstruction (Saso et al., 2019). Gliomatosis peritonei (GP) is a condition often found alongside a diagnosis of immature teratoma and presents with mature glial tissue implants within the peritoneum (Saso et al., 2019, Li et al., 2016). Only approximately 100 cases of GP have been reported since 2016 (Bajracharya et al., 2021).

Synchronous GTS and GP is a scarcely reported phenomenon with, to our knowledge, only six previously published case reports or descriptions (Nitecki et al., 2023, Joshua et al., 2022, Nasfy et al., 2023, Małgorzata et al., 2021, Li et al., 2022, Mrabti et al., 2011; 2011.). To expand on existing literature and better understand the clinical and radiologic course, we describe the case of a young woman diagnosed with extra pelvic GTS and GP approximately ten months after primary surgery and seven months after completion of adjuvant chemotherapy.

## 2 Case

**Fig. 1A-C:**
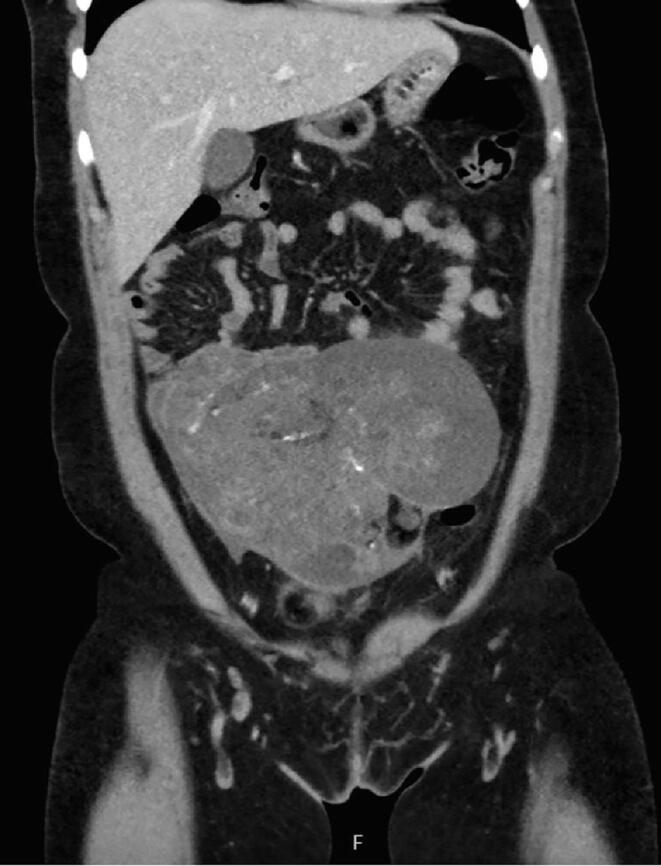

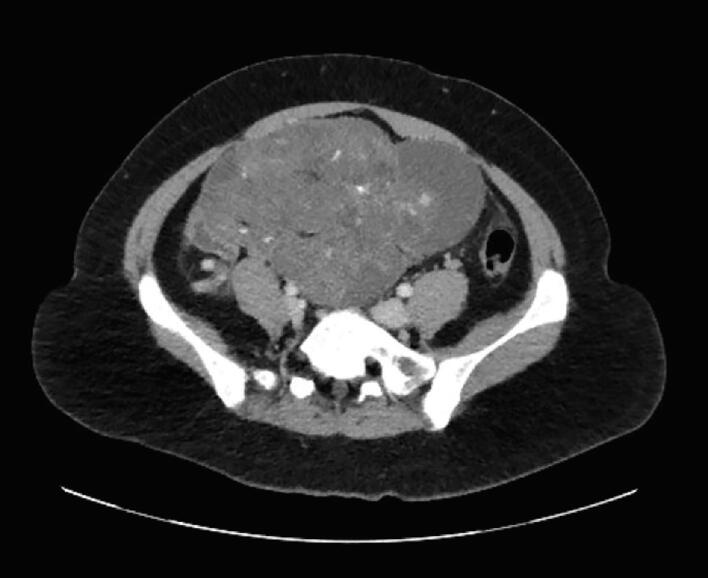

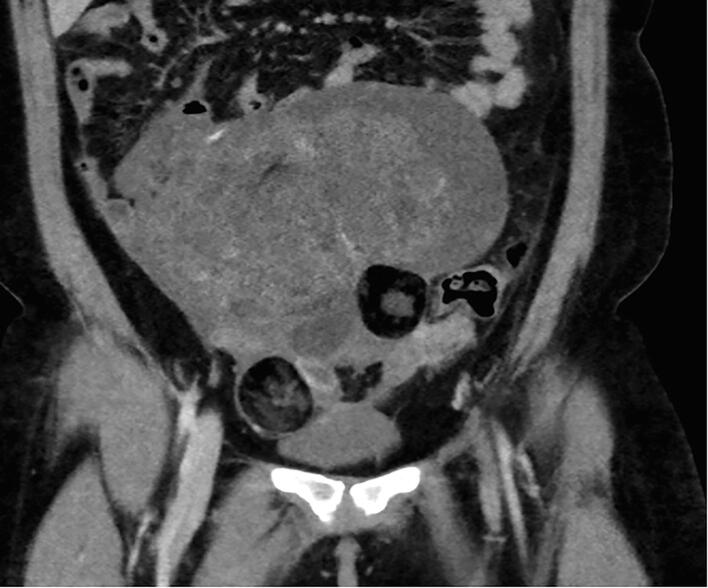
26-year-old female with lower abdominal pain, found to have small bilateral mature teratomas measuring approximately 4 cm, and large 18 cm left adnexal mass that is predominantly cystic and solid, with small scattered fatty components and tiny scattered calcification concerning for immature teratoma.

A 26-year-old G1P1001 presented in March of 2023 with three weeks of worsening right lower quadrant abdominal pain and a recent diagnosis of chlamydia two weeks prior. She had not been compliant with doxycycline and presented with WBC count of 13.9 cells*/*μl. In the emergency department, a transvaginal ultrasound demonstrated a 18.6 cm mixed cystic and solid pelvic mass concerning for tubo-ovarian abscess given recent infection and therefore the patient was initiated on antibiotics. Subsequently, a CT scan was obtained demonstrating bilateral mature teratomas and a 17.6 x 11.1 x12.6 cm mixed solid and cystic multilobulated pelvic mass (Fig. 1A-1C). Her tumor markers at that time were remarkable for LDH of 303 U/L, AFP of 488.3 ng/mL, and CA125 of 81 U/mL.

The patient was subsequently referred to gynecologic oncology. One week after her hospital visit, she underwent exploratory laparotomy, left salpingo-oophorectomy, right ovary cystectomy, and appendectomy. Her intraoperative findings were significant for a complex 17 cm left ovarian mass and an 8 cm right ovarian mass with frozen sections consistent with mature teratoma with focal immature teratoma. Her final pathology of the left mass demonstrated Stage IA mixed malignant germ cell tumor (>99 % G2 immature teratoma, <1% yolk sac tumor), and her right mass was a mature cystic teratoma.

The patient completed three cycles of Bleomycin, Etoposide, and Cisplatin in June of 2023. She underwent surveillance with alternating transvaginal ultrasound and CT scan every 3 months, and tumor markers every 3 months. She was without evidence of disease by markers and imaging until seven months later when CT imaging revealed a 3.5 x 4.2 x 2.8 cm mass within the right anterior abdominal mesentery containing some stranding, fluid and a calcification of uncertain significance (Fig. 2A-B, Fig. 2CA-2B). No new lymphadenopathy was identified. The LDH, AFP, and CA125 remained normal range at 160 U/L, 2.7 ng/mL, and 25 U/mL, respectively.

**Fig. 2A-B:**
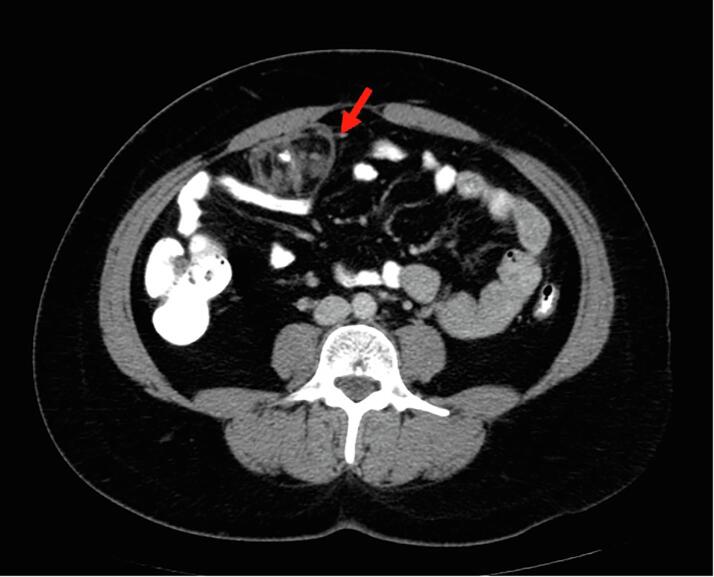

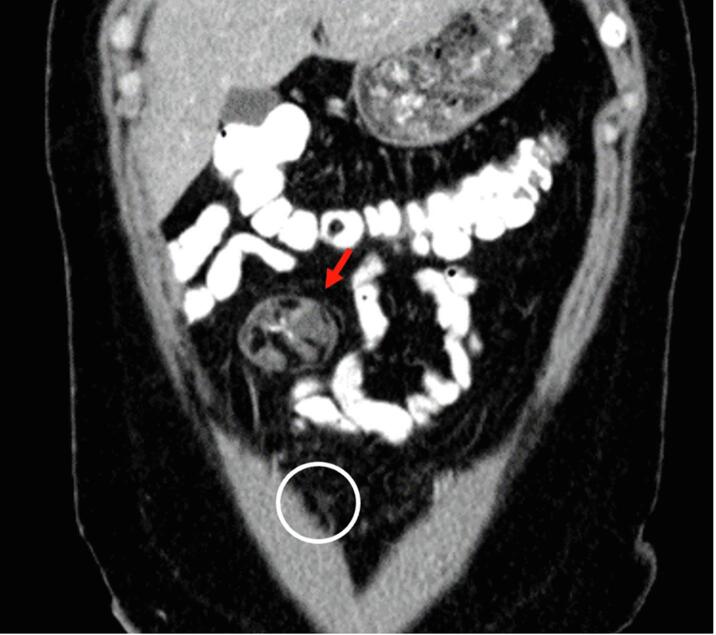
Ten months after surgical resection of immature teratoma, and seven months after chemotherapy completion the restaging CT demonstrates a 5 cm mass (red arrow), predominantly fatty with single calcification, with similar appearance to mature teratoma demonstrated in the CT performed at presentation. There is also trace peritoneal stranding (white circle), which is difficult to distinguish from benign process such as non-specific scarring, trace fluid, early/subtle malignant involvement of peritoneum, or gliomatosis peritonei. (For interpretation of the references to colour in this figure legend, the reader is referred to the web version of this article.)

The patient underwent exploratory laparotomy, mesenteric tumor resection (5 x 4 x 3.5 cm), and partial omentectomy with resection of miliary omental implant one year after initial presentation in March of 2024. Pathology of the omental mass was consistent with mature teratoma. No immature components were identified. The omentectomy pathology was benign gliomatosis. Patient was presented at tumor board with a plan for surveillance with alternating transvaginal ultrasound and CT scan every 3 months, and tumor markers every 3 months. There was no plan for initiation of adjuvant therapy.

At the time of this report, the patient remains without evidence of recurrent tumor nine months after her exploratory surgery.

## 3 Discussion

Synchronous GTS and GP remains a rare phenomenon and is primarily reported as case reports or case series (Li et al., 2016, Nitecki et al., 2023, Joshua et al., 2022, Nasfy et al., 2023, Małgorzata et al., 2021, Mrabti et al., 2011; 2011., Semiz et al., 2022, Gocht et al., 1995). Few papers analyze GP in greater than 5 cases (Robboy and Scully, 1970, Müller et al., 2002, Bentivegna et al., 2015, Harms et al., 1989, Norris et al., 1976, Liang et al., 2015, Yoon et al., 2012). Reviews have demonstrated that GP is overall associated with a favorable prognosis, frequently remaining a quiescent entity; however, often related to recurrence in patients with immature ovarian teratoma (Yoon et al., 2012).

The incidence of GTS remains difficult to estimate; however, it has been quoted as high as 40 % (Bentivegna et al., 2015, Yoon et al., 2012, Van Nguyen et al., 2016). For this reason, clinical suspicion should be high in patients found to have recurrent masses after treatment of immature teratoma. Previous studies have demonstrated a wide spectrum of diagnosis with some studies identifying GTS as early as three months and others greater than two years post-operatively (Li et al., 2016). At least two published reviews report a mean 15–27 months on average for presentation of GTS. We present a case of GTS and GP arising 12 months after primary surgery.

Numerous case reports have identified growing teratoma syndrome most commonly affecting pelvic organs, as well as few reporting extra pelvic location (Saso et al., 2019), such as the upper abdomen, retroperitoneal, and liver/diaphragm/paracecal (Altinbas et al., 2013, Dewdney et al., 2006, Tangjitgamol et al., 2006). We present a case of extra pelvic metastases with a five-centimeter mass identified in the mesentery on imaging. This addition to the existing literature raises the importance of both pelvic and abdominal imaging as part of the surveillance protocol for immature teratomas.

Identification of GTS on imaging is key to identification of recurrence. On ultrasound, the mature teratoma of GTS often presents as a unilocular cystic mass with some mural components. The sebaceous component may present as diffusely or partly echogenic mass with posterior attenuation resulting in the tip of the ice-berg sign (Fig. 2A-B, Fig. 2CC).

**Fig. 2C:**
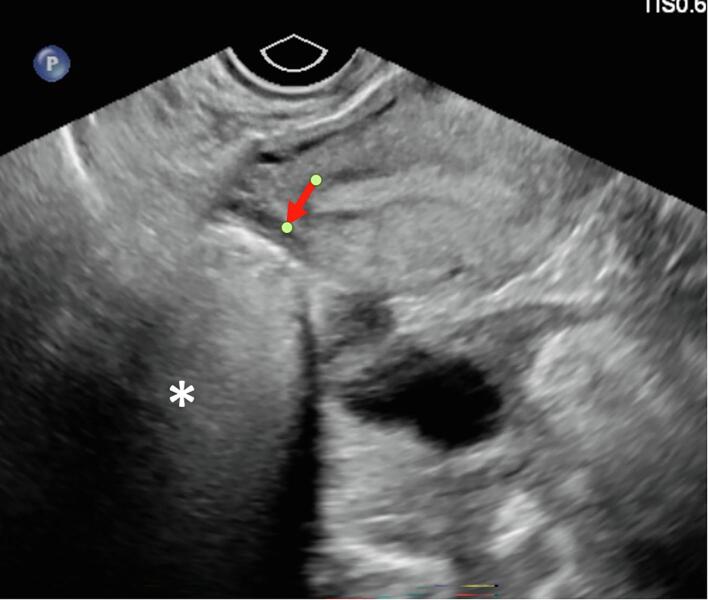
26-year-old female with lower abdominal pain, on ultrasound had bilateral mature teratomas measuring approximately 4 cm. Transvaginal ultrasound demonstrates hyperechoic mass (red arrow) with posterior attenuation (white *), known as the tip of the iceberg sign. Posterior and superior to the uterus fundus, solid mass with cystic component is partially visualized, demonstrating large pelvic mass with mixed solid and cystic component with minimal scattered fat better identified on CT.

It may contain focal dense shadowing from one or few calcified/ossified components, or with fluid/fluid levels. Hair in the cyst may result in dot dash pattern with-in the sebaceous cyst. A fatty component may be observed floating to the non-dependent location within a cystic mass, an uncommon but pathognomonic finding of mature teratoma. On CT, mature teratomas are easily identified when containing fat, demonstrating the characteristic low attenuation, sometimes as fat-fluid level, others as Rokitansky protuberance (dermoid plug) within a cystic mass. It may be associated with single or few chunky/tooth-like calcification or no calcification (Fig. 1A-1C). MRI best delineates the soft tissue characteristics of the fatty component and its exact location, number, size and relationship with adjacent structures, along with signs of complication. Of note, 6 % of mature teratomas are without fat component, and therefore, are indistinguishable from other non-specific benign cystic lesions of the ovary. On PET/CT, it demonstrates little or no hypermetabolic activity (Sasahara et al., 2022, Kikawa et al., 2011). Immature teratoma compared to mature teratoma have much higher proportion of solid components, and smaller fatty components, and if calcifications are present, they are much smaller and scattered.

Gliomatosis peritonei is a peritoneal involvement of benign mature glial implants, most commonly seen associated with ovarian teratomas, and rarely associated with ventriculoperitoneal shunts (Lobotesis et al., 2009). It is not distinguishable from peritoneal implant of recurrent immature teratomas, or other benign or malignant process.

Growing teratoma syndrome with synchronous gliomatosis peritonei is an infrequently described event. This case report adds to the existing literature and expands upon current knowledge by specifically highlighting the importance of completing both pelvic and abdominal imaging to assess for this phenomenon. When masses are identified on imaging, there should be a high index of suspicion for GTS in a patient who has been treated for immature teratoma. Once identified, biopsy or surgical excision is recommended to exclude recurrent immature teratoma avoid unnecessary chemotherapy and prevent mechanical obstruction and future complications.

## 4 Study approval statement

This study protocol was reviewed and approved by the University of California Institutional Review Board.

## 5 Consent to publish statement

Written informed consent was obtained from the patient for publication of this case report. A copy of the written consent is available for review by the Editor-in-Chief of this journal on request.

## 6 Funding sources

The study was not supported by any sponsor or funder.

## CRediT authorship contribution statement

**Brittany File:** Writing – review & editing, Writing – original draft, Investigation, Conceptualization. **Sonia Lee:** Writing – review & editing, Writing – original draft, Conceptualization. **Robert Bristow:** Writing – review & editing, Conceptualization.

## Declaration of Competing Interest

The authors declare that they have no known competing financial interests or personal relationships that could have appeared to influence the work reported in this paper.
